# Respiratory‐Resolved Isotropic 3D T_1_ Mapping of the Carotid Vessel Wall With B_1_ ^+^ Correction (CARISMAT1C)

**DOI:** 10.1002/mrm.70587

**Published:** 2026-09-04

**Authors:** Isabel Montón Quesada, Céline Hirsch, Pauline Calarnou, Jean‐Baptiste Ledoux, Jérôme Yerly, Christopher W. Roy, Augustin C. Ogier, Ruud B. van Heeswijk

**Affiliations:** ^1^ Department of Radiology Lausanne University Hospital (CHUV) and University of Lausanne (UNIL) Lausanne Switzerland; ^2^ CIBM Center for Biomedical Imaging Lausanne Switzerland

**Keywords:** 3D T_1_ mapping, B_1_^+^ correction, carotid artery, free‐running, respiratory‐resolved

## Abstract

**Purpose:**

To develop, optimize, and characterize a respiratory motion‐resolved free‐running isotropic 3D carotid vessel wall T_1_ mapping technique named CARISMAT1C.

**Methods:**

An inversion‐recovery gradient‐echo free‐running pulse sequence with interleaved double flip‐angle (2FA) was implemented, and an extended‐phase‐graph dictionary was used to map the T_1_ relaxation time. In a phantom, the T_1_ accuracy was compared to that of a single‐flip‐angle (1FA) variant, the inversion‐recovery spin‐echo reference, and clinical routine MOLLI, while the T_1_ precision of several phyllotaxis‐based radial trajectories was compared. The most precise sequence was used in 11 healthy volunteers (25 ± 3Y, 3F). A synthetic 3D gray‐blood image was reconstructed from the source images and used to delineate the internal, external, and common carotid arteries. T_1_ values across artery sections were compared to those of MOLLI.

**Results:**

In the phantom, the 2FA variant had higher T_1_ agreement with the IR‐SE reference than 1FA or MOLLI. The top‐to‐bottom phyllotaxis trajectory with golden‐step shuffling was the most precise and was retained for in vivo scans. End‐expiratory carotid vessel wall T_1_ values (1185 ± 42 ms) were similar to those obtained with MOLLI (1162 ± 84 ms, *p* = 0.37) and showed less intersubject variability and high interscan repeatability. No significant differences were found across arterial segments in the vessel wall T_1_ values.

**Conclusion:**

CARISMAT1C had high T_1_ accuracy in phantoms and high T_1_ precision in vivo in different sections of the carotid artery tree.

## 1 Introduction

Stroke is the world's second most prevalent cause of mortality and is the leading preventable cause of disability worldwide [1]. One of its main causes is the rupture of atherosclerotic plaques in large blood vessels like the carotid artery. Despite the availability of several therapies to prevent plaque rupture, there is a lack of diagnostic techniques that enable the early and accurate detection of plaque vulnerability (i.e., the risk of plaque rupture). This diagnostic gap increases the risk of unexpected plaque rupture and subsequent stroke. Besides clinical symptoms, current treatment decisions are mostly based on the degree of stenosis of the carotid artery, for which techniques such as computed tomography angiography, ultrasound and magnetic resonance angiography are used. However, the vulnerability is more accurately characterized by specific pathological characteristics such as a thin fibrous cap, hemorrhage, calcification, and a lipid‐rich necrotic core [2]. Although multi‐contrast protocols are increasingly being used for risk stratification of carotid atherosclerotic plaque [3], they require the acquisition of multiple contrast‐weighted images, resulting in long scan times and potential image misregistration, and are mostly limited to academic centers, as they have not been incorporated to clinical guidelines.

T_1_ mapping of the carotid artery can be used to non‐invasively and quantitatively assess the composition of the vessel wall and plaque components in one scan [4, 5]. Qiao et al., for example, quantified plaque vulnerability with T_1_ mapping and validated it through histology, showing the potential of T_1_ mapping for evaluating plaque components and rupture risk [4]. However, T_1_ estimations can be easily biased by inhomogeneities in the radiofrequency transmit field (B_1_^+^), especially at higher magnetic field strengths. To correct these inhomogeneities, B_1_^+^ maps can be separately acquired using spoiled gradient‐recalled echo (GRE) images where the flip angle is varied. Alternatively, the B_1_^+^ field can be mapped intrinsically from acquired data without a separate acquisition to ensure co‐registration with the T_1_ maps [6]. In inversion‐recovery T_1_ mapping, the B_1_^+^ map can be most straightforwardly generated by varying the flip angle after each inversion pulse in an interleaved fashion. As a result, the B_1_^+^ map can be obtained as the voxel‐wise ratio β between the nominal flip angle α and the true flip angle $\alpha_{\text{true}}$ at position **r**:

(1) $$\alpha_{\text{true}}\left(r\right)=\beta \left(r\right)\alpha$$

As parametrical mapping implies a voxel‐wise fitting across multiple source (or contrast) images, any misalignment between these images can lead to inaccurate T_1_ estimates. This underscores the importance of compensating motion in the image reconstruction [7]. Several sources of motion can confound neck T_1_ mapping, such as bulk motion, swallowing, respiration, and carotid pulsation [8]. Swallowing motion is characterized by a high amplitude and low frequency signal on physiological monitoring. Respiratory motion perturbations exhibit intermediate amplitude and frequency, and cardiac or pulsatile motion is a perturbation with low amplitude and high frequency. Since breathing‐induced motion causes the highest periodic displacement of the carotid arteries in neck MR images, this work focuses on resolving this motion.

Therefore, the goal of this work was to develop a T_1_ mapping technique that simultaneously addresses the B_1_^+^ and respiratory motion confounders. This approach (named CARISMAT1C for **CA**rotid **R**espiratory‐resolved **IS**otropic **M**ulti‐**A**ngle **T1** mapping with B_1_^+^ **C**orrection) combines a GRE acquisition with two flip angles for B_1_^+^ correction and a self‐gated respiratory‐resolved reconstruction to generate co‐registered T_1_ and B_1_^+^ maps. This framework was evaluated in numerical simulations, the ISMRM‐NIST phantom, and in healthy volunteers.

## 2 Methods

This study was performed on a clinical 3T MR scanner (MAGNETOM PrismaFit, Siemens Healthcare, Erlangen, Germany).

### 2.1 Phantom Experiments

#### 2.1.1 Sequence Design

Three pulse sequences were compared in the ISMRM‐NIST phantom [9] to quantify the effect of B_1_^+^ correction: (1) a GRE free‐running single‐flip‐angle (1FA) sequence; (2) a GRE free‐running dual‐flip‐angle (2FA) sequence; and (3) modified Look‐Locker inversion (MOLLI), a clinical routine ECG‐triggered pulse sequence for T_1_ mapping [10]. For the design of the free‐running T_1_ mapping sequence, a free‐running framework [11, 12] was modified by including non‐selective hyperbolic secant adiabatic inversion pulses (Figure 1A) followed by 432 readouts ordered in a 3D golden‐angle phyllotaxis radial trajectory [13], which resulted in a 2572 ms inversion pulse spacing. Each of the interleaves (18 in total) consisted of 24 readouts following a spiral pattern. Acquisition parameters included: acquired and reconstructed isotropic voxel size (0.78 mm)^3^, isotropic field of view (FOV) 200 mm, bandwidth 501 Hz/pixel, repetition time (TR)/echo time (TE) 5.82/2.64 ms, time between the inversion pulse and the first excitation pulse T_gap_ = 33.3 ms, longitudinal recovery time T_ex_ = 4.8 ms (Figure 1A) and total scan time = 9 min 58 s, for both 1FA and 2FA sequences. For the 1FA sequence the flip angle was set to 6°. In the 2FA sequence, the flip angle was varied in an interleaved fashion (α_1_ = 3°, α_2_ = 10°) after each inversion pulse, ensuring that k‐space lines acquired with different flip angles were subject to comparable motion. This contrasts with sequential acquisition schemes, in which half of the data are acquired with one flip angle followed by the remaining half with the other, potentially introducing motion‐related biases. The 2D MOLLI sequence was acquired with pixel size (0.8 mm)^2^, slice thickness 8 mm and flip angle 12°. T_1_ values obtained with these sequences were correlated with the values of a reference 2D inversion‐recovery spin‐echo (IR‐SE) sequence with inversion times TIs = 21–50–100–250–1000–2000–4000 ms. The regional T_1_ root‐mean‐squared error (RMSE) was computed.

**FIGURE 1:**
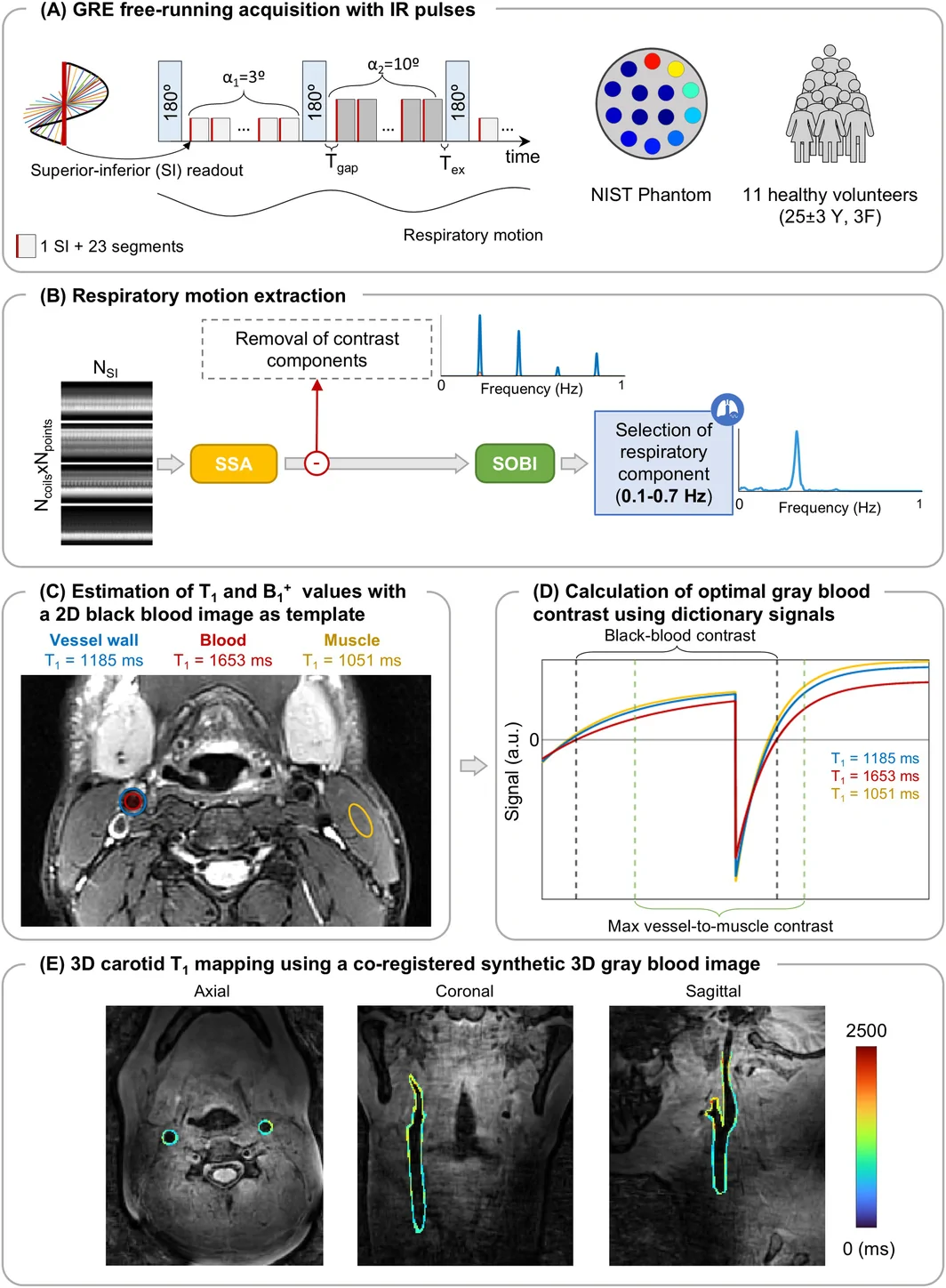
Free‐running 2FA 3D T_1_ mapping framework. (A) Interrupted free‐running data acquisition with interleaved dual‐flip angle technique. (B) Respiratory signal extraction is performed by applying SSA and SOBI to the superior–inferior (SI) projections 2D matrix. (C) Reference delineation of the carotid vessel wall, blood and muscle to obtain T_1_ values for (D) the calculation of a gray‐blood contrast from combined blood nulling and maximum vessel‐to‐muscle contrast. Inflow effects were not explicitly modeled in the blood signal evolution. (E) Reconstruction of a 3D gray‐blood image overlaid with the 3D T_1_ map of the carotid vessel wall.

To compare the B_1_^+^ map obtained with the 2FA sequence, a separate routine B_1_^+^ map was acquired with the following parameters: pixel size 0.8 × 0.8 mm^2^, slice thickness 6 mm, slice spacing 12 mm, and FOV 130 × 160 mm^2^. Correlation of the sphere B_1_^+^ values between maps was evaluated with the Pearson correlation coefficient *r*.

#### 2.1.2 Trajectory Optimization

The standard half‐phyllotaxis trajectory (HP, Figure 2A) [13] has been proven to be robust to motion and improve image quality. However, phase and magnitude artifacts can be introduced due to gradient imperfections during data sampling. In addition, binning‐related artifacts during the motion and contrast‐resolved reconstruction can appear due to sequential reordering of the polar angles of the spiral interleaves that create coherent undersampling patterns [14, 15]. To test the hypothesis that dealing with both the phase‐ and binning‐related artifacts would improve T_1_ precision, 4 variations of the radial phyllotaxis trajectory were compared: (1) HP (Figure 2C); (2) HP with golden‐step [15] shuffling of the interleaves along the polar direction based on the golden ratio to improve the uniformity of the k‐space density distribution per bin to prevent binning‐related artifacts (HP‐GS, Figure 2E); (3) a top‐to‐bottom (T2B, Figure 2B,D) phyllotaxis trajectory [16, 17] to minimize phase artifacts by starting sampling data from both k‐space hemispheres; and (4) T2B with golden‐step shuffling (T2B‐GS, Figure 2F). As a measure of incoherence of the trajectory variations, we computed the side‐lobe‐to‐peak ratio (SPR) [18] and the ratio of the standard deviation of the side lobe magnitudes to the main peak magnitude (S_SD_PR) [19] of the point spread function (PSF) for each of the contrast bins. Agreement and RMSE of the resulting T_1_ values with IR‐SE reference values were calculated. The trajectory with highest agreement was retained and used to map the carotid artery in vivo.

**FIGURE 2:**
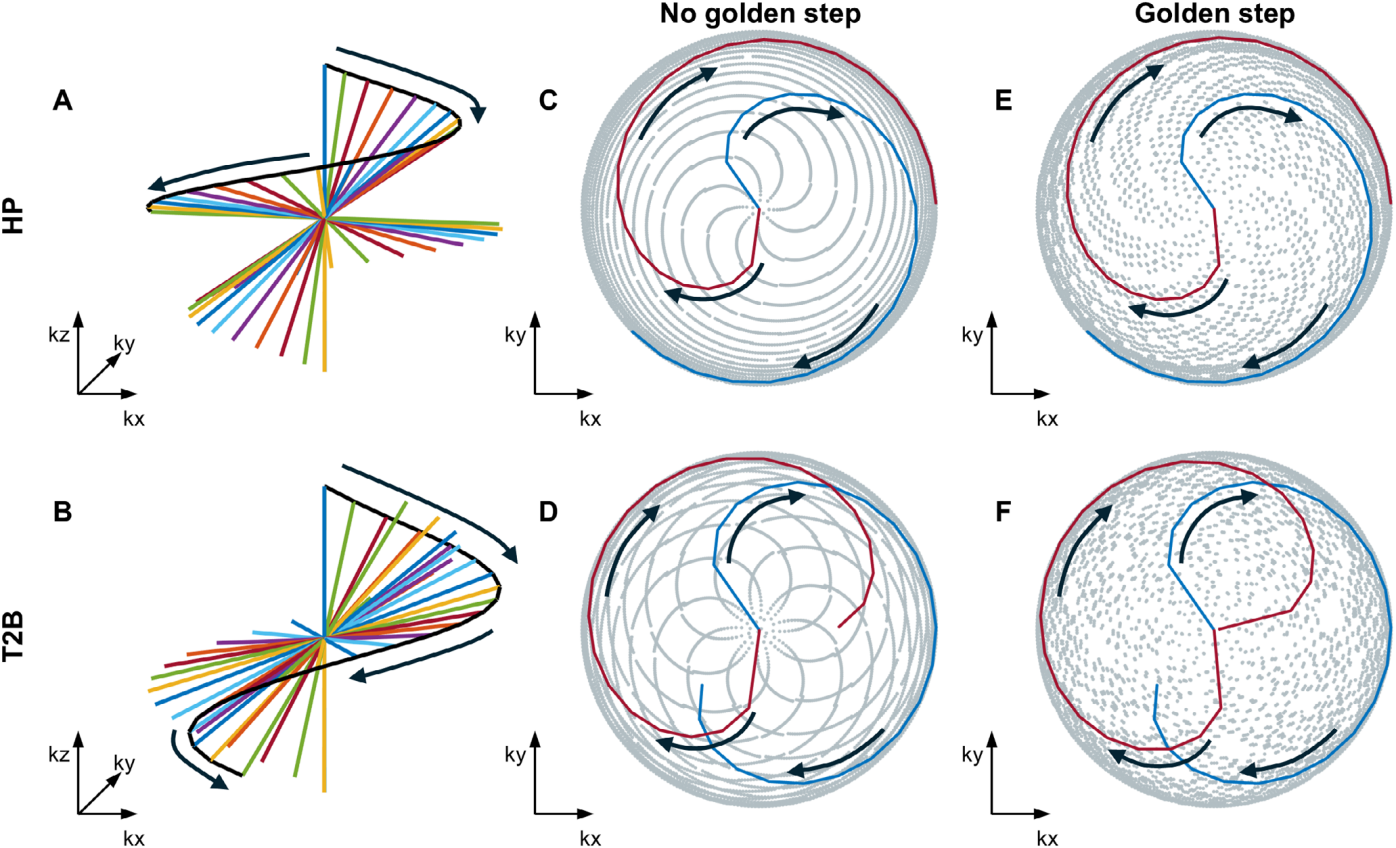
(A) One spiral interleave (black line) with 24 readouts corresponding to the half phyllotaxis (HP) trajectory. (B) One spiral interleave with 24 readouts corresponding to the top‐to‐bottom phyllotaxis (T2B) trajectory. (C,D) k‐space distribution of the 15th contrast bin when the golden‐step shuffling is not applied. Clear spiral‐like patterns and k‐space point accumulation can be observed in both HP and T2B cases. (E,F) Same bin but in this case the golden‐step shuffling is implemented which clearly reduces point accumulation and improves the uniformity of the sampling distribution.

#### 2.1.3 Static Image Reconstruction

To reduce phase errors due to the inversion pulse, coil sensitivities were computed using the Walsh method [20] and estimated from the last 80% of readouts acquired following the 10° flip angle IR pulse (corresponding to 38% of the total number of readouts).

The 1FA and 2FA k‐space data were sorted in 8 contrast bins per flip angle (8 for the 1FA and 16 for the 2FA) to account for the flip angle variation. A k‐t sparse SENSE compressed sensing algorithm [21, 22] was used to reconstruct the static images by solving the following optimization problem:

(2) $$\hat{x\;}=\underset{x}{\text{argmin}}\left({\left\|\mathrm{FCx}-y\right\|}_{2}^{2}+\lambda_{s}{\left\|\nabla_{s}x\right\|}_{1}+\lambda_{T1}\sum_{i}{\left\|H_{i}x\right\|}_{*}\right),$$

where $\hat{x}$ is the reconstructed image, F the non‐uniform Fourier transform operator, C the coil sensitivity maps, and y the k‐space raw data. $\nabla_{s}$ is the first‐order differential operator along the spatial dimension and $H_{i}$ is the operator that extracts the *i*‐th spatial patch of size 5 × 5 × 5 pixels from x and forms a Casorati matrix across the contrast dimension. ${\left\|\cdot \right\|}_{2}^{2}$, ${\left\|\cdot \right\|}_{1}$ and ${\left\|\cdot \right\|}_{*}$ are the L2, L1 and nuclear norms, respectively, and correspond to the data‐fidelity term, the spatial sparsity regularization through total variation, and local low‐rank constraint. The spatial total variation and local low‐rank regularization weights $\lambda_{s}$ and $\lambda_{T1}$ were empirically set to 0.05 and 0.5 respectively. The optimization problem was solved using the Alternating Direction Method of Multipliers (ADMM) algorithm [23] with 10 iterations. With this iterative reconstruction, all 3D radial lines have unique trajectories, so all lines improve the SNR and contribute to the precision of the T_1_ and B_1_^+^ estimation.

A Linux workstation with two 24‐core CPUs (Intel Xeon Gold 6248R; Intel, Santa Clara, CA, USA), 1.5 TB of RAM, and dual NVIDIA RTX A6000 GPUs (Nvidia, Santa Clara, CA, USA) was used to perform offline image and map reconstruction in MATLAB R2024a (The MathWorks, Natick, MA, USA).

#### 2.1.4 Dictionary Generation and Fitting

Co‐registered T_1_ and B_1_^+^ maps were obtained using dictionary matching after extended phase graph (EPG) simulations [24]. The set of parameters simulated for T_1_ was (10:2:4000) ms and (0:0.05:2.5) for B_1_^+^. The T_2_ value was set to 50 ms, which corresponds to healthy carotid vessel wall [25]. The dictionary was generated only once per free‐running sequence as the acquisition timing did not depend on cardiac or respiratory triggers. Dictionary matching was performed by maximizing the dot product between the simulated signals and the voxel‐wise signal evolution from the source images. To match the number of reconstructed source images (i.e., contrast dimension), the simulated signal evolution was divided into 8 and 16 bins for the 1FA and 2FA techniques, respectively, and the signal contributions within each bin were averaged.

### 2.2 In Vivo Imaging

#### 2.2.1 Study Cohort and Acquisition Protocol

The in silico optimized free‐running sequence that yielded the best results was retained for scanning in vivo. Thus, the 2FA sequence with the optimized trajectory was used to map the carotid artery T_1_ values in 11 healthy volunteers (25 ± 3 years, 3 females). All experiments were approved by the local ethics committee (Ethics Committee of the Canton of Vaud (CER‐VD) number 2021–00697), and written and informed consent was obtained from all participants.

MOLLI was included in the in vivo protocol as a clinical routine comparator. In addition, a pulse‐oximeter‐triggered T2‐weighted with fat saturation RF pulses 2D turbo spin echo black‐blood image was acquired with pixel size 0.8 × 0.8 mm^2^, slice thickness 3 mm and total acquisition time 2 min.

#### 2.2.2 Respiratory Motion Extraction and Image Reconstruction

The repeated superior–inferior (SI) readouts (Figure 1B) were used for respiratory motion extraction. After computing their one‐dimensional Fourier transform for all individual RF coil elements, they were concatenated in a 2D matrix (Figure 1B). Singular spectrum analysis (SSA) [26] was then applied to remove the signal variations caused by the IR module. Afterwards, second‐order blind identification (SOBI) [11, 27] was used to extract the respiratory motion component. The accuracy of the self‐gated respiratory component was validated with a respiratory bellows signal recorded during the scan by computing their Pearson correlation coefficient.

The self‐gated respiratory component was used to order the continuously acquired k‐space data in respiratory bins. Binning in the respiratory dimension was performed by dividing the respiratory signal into 4 equally populated phases [28] (from end‐expiration to end‐inspiration) to separate the acquisition into the different motion states of the respiratory cycle. Binning in the contrast dimension was performed as described above. The scan efficiency (defined as the percentage of total scan time dedicated to data acquisition) was computed.

For image reconstruction, Equation (2) was adapted to include the respiratory dimension:

(3) $$\hat{x\;}=\underset{x}{\text{argmin}}\left({\left\|\mathrm{FCx}-y\right\|}_{2}^{2}+\lambda_{s}{\left\|\nabla_{s}x\right\|}_{1}+\lambda_{r}{\left\|\nabla_{r}x\right\|}_{1}+\lambda_{T1}\sum_{i}{\left\|H_{i}x\right\|}_{*}\right),$$

where $\nabla_{r}$ is the first‐order differential operator along the respiratory dimension. $H_{i}$ denotes the operator that extracts the *i*‐th spatial patch of size 5 × 5 × 5 × 4 pixels from x and forms a Casorati matrix across the combined contrast and respiratory dimensions. The spatial and respiratory regularization weights $\lambda_{s}$ and $\lambda_{r}$ were both empirically set to 0.01 [29].

#### 2.2.3 Image Analysis

To initially calibrate the tissue characterization, the 2D black‐blood image was used as a guide to delineate anatomy and thus to obtain representative T_1_ values to generate a 3D gray blood image from the mapping data. To this end, the inner and outer vessel wall and blood pool contours of the common carotid artery (CCA) were manually delineated, and a region of interest (ROI) was drawn in the sternocleidomastoid muscle. These segmentations were then copied to a matching reformatted 2D slice from the estimated T_1_ and B_1_^+^ maps (Figure 1C) of which the in‐plane resolution was interpolated to (0.4 mm)^2^. The T_1_ and B_1_^+^ values in the ROIs were used to identify each tissue's representative dictionary entry and to generate a 3D synthetic gray‐blood image using an adapted narrow‐band k‐space‐weighted image contrast (KWIC) filter [30]. This was achieved by combining the k‐space lines acquired at the blood nulling point and the maximal vessel‐to‐muscle contrast point (Figure 1D,E). The number of readouts per contrast point was set to 20 fully sampled readouts and 120 readouts with only samples from the higher frequencies of the k‐space (Figure 3). These values were empirically chosen based on qualitative assessment of vessel wall contrast and sharpness. Gray‐blood images were denoised using BM4D, with a Rician noise model with the noise standard deviation automatically derived from the images [31]. Once the average tissue T_1_ values were established, a gray‐blood image could be generated directly from the raw data and the separate black‐blood image was no longer needed.

**FIGURE 3:**
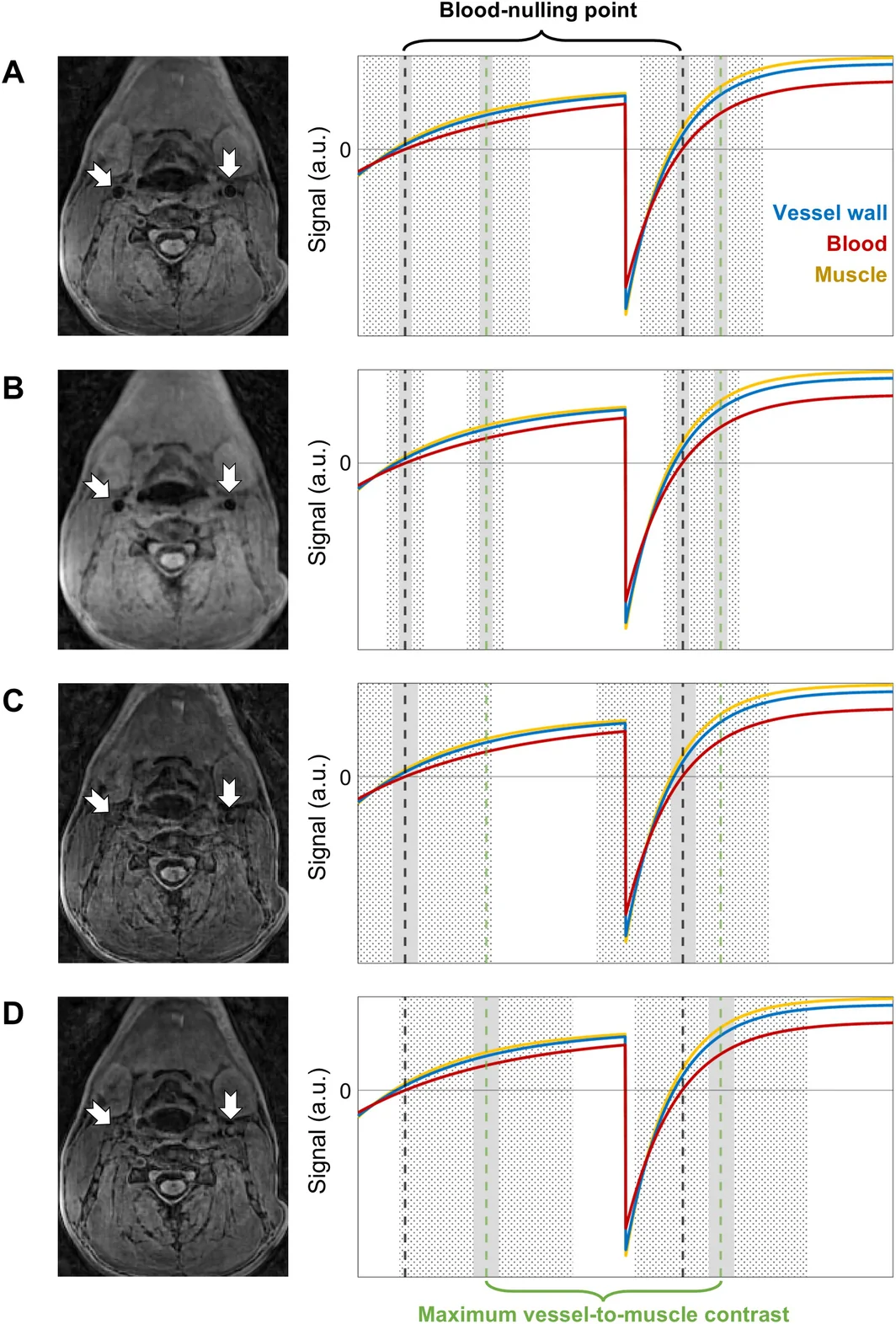
Gray‐blood image generation. (A) Gray‐blood image with 20 contrast lines (gray‐shaded area) and 120 resolution lines (dotted area) per recovery curve. The vessel wall can clearly be discerned (white arrows). (B) Gray‐blood image when using only 40 resolution lines. All structures are more blurred. (C) Generated image when only using the black‐blood contrast lines. The vessel wall is not well‐defined. (D) Generated image when only using the maximum vessel‐to‐muscle contrast lines. Bright blood can be observed inside the carotid arteries.

T_1_ values from the reformatted slice at the vessel wall and muscle were compared to those obtained with MOLLI.

Repeatability of vessel wall and muscle T_1_ measurements from 2FA and MOLLI maps was evaluated by scanning a subset of 5 volunteers (27 ± 4 years, 1 female) twice on separate days.

### 2.3 Statistical Analysis

Phantom 1FA, 2FA and MOLLI T_1_ values were compared to the IR‐SE values using linear regression and Bland–Altman analyses to evaluate correlation and agreement, respectively. Normality of the data was assessed using the Shapiro–Wilk test. Two‐sided paired Student's *t*‐tests were used to compare normally distributed groups and Wilcoxon signed‐rank tests were used in case of non‐normally distributed data. The coefficient of variation (CoV) was computed for the scan‐rescan analysis, and Bland–Altman analyses were conducted, as well as Student's *t*‐tests to compare the bias between scans.

T_1_ values for the vessel wall of the CCA, internal (ICA), and external (ECA) carotid arteries were compared using repeated measures ANOVA, followed by post hoc Tukey analysis. All results are reported as mean ± standard deviation for normal data and median (interquartile range) for non‐normal data. A *p*‐value lower than 0.05 was considered statistically significant.

## 3 Results

### 3.1 Phantom Studies

#### 3.1.1 B_1_ ^+^ Correction

Phantom spheres with IR‐SE T_1_ values between 100 ms and 1500 ms were analyzed (Figure 4A–D). When examining the routine B_1_^+^ map, a pattern of increasing values from the periphery to the center of the phantom could be observed (Figure 4E). A similar pattern could also be seen in the B_1_^+^ map generated with the 2FA technique (Figure 4F), with a correlation with the reference technique *r* = 0.86. The estimated 2FA T_1_ values showed strong linear correlation (Figure 5A) and lower RMSE (RMSE_2FA_ = 13.7 ms) compared to 1FA (RMSE_1FA_ = 21.9 ms) and MOLLI (RMSE_MOLLI_ = 16.0 ms). Bland–Altman analysis (Figure 5B) demonstrated that 2FA T_1_ values had narrower confidence intervals (−44.3 to 56.6 ms) than those from 1FA (−187.3 to 108.2 ms) and MOLLI (−36.8 to 73.8 ms). In addition, 2FA had the lowest bias (6.1 ms) among the three techniques (−39.6 ms for 1FA and 18.5 ms for MOLLI), indicating the highest agreement with the IR‐SE reference.

**FIGURE 4:**
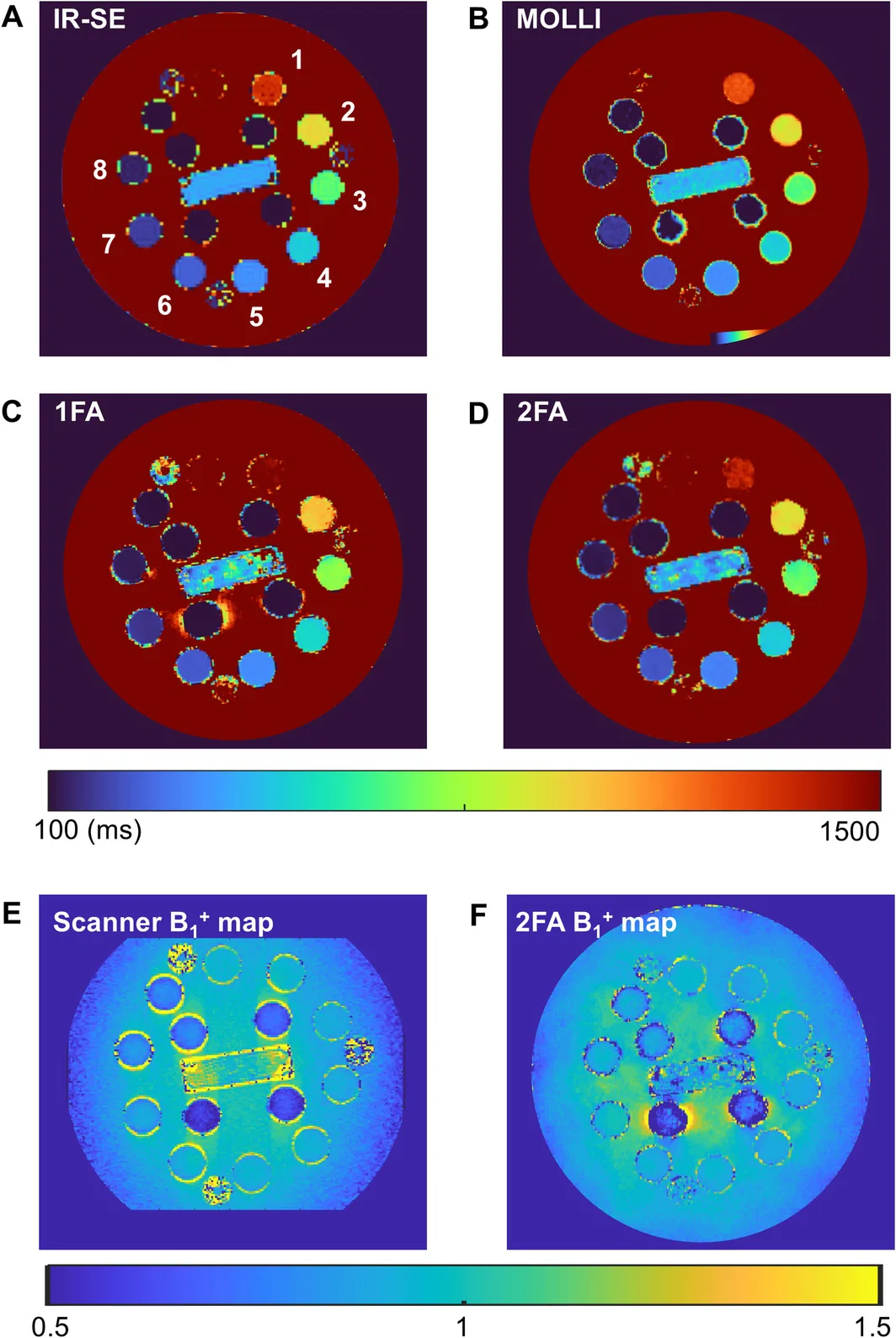
T_1_ and B_1_^+^ maps in the ISMRM‐NIST phantom. (A) Reference IR‐SE T_1_ map. (B) MOLLI T_1_ map. Only numbered spheres are considered for subsequent analysis. (C) 1FA T_1_ map. (D) 2FA T_1_ map; note the decrease in minor artifacts compared to the 1FA T_1_ map. (E) Routine B_1_^+^ map and (F) B_1_^+^ map generated with the 2FA technique where a pattern of increasing values from the periphery to the center can be observed.

**FIGURE 5:**
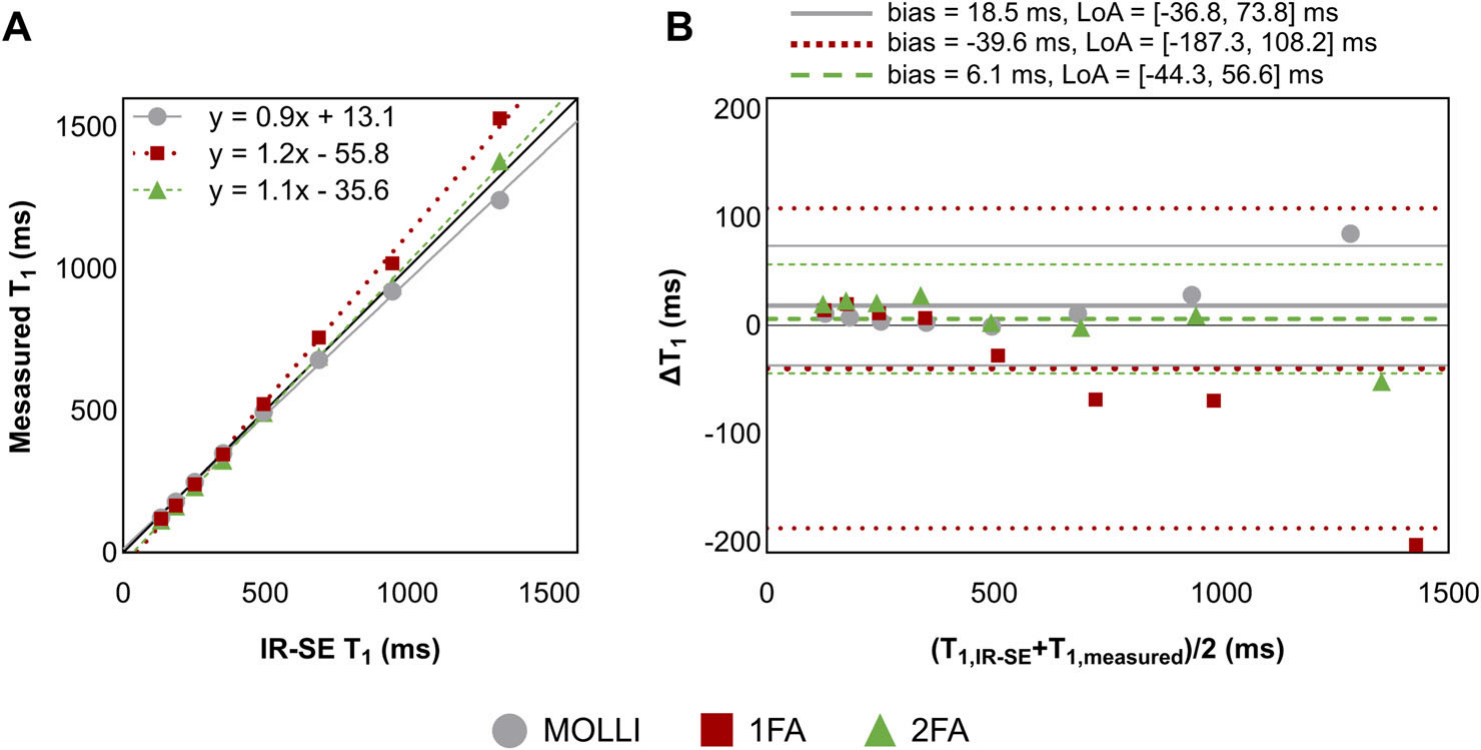
Accuracy and precision of the proposed techniques in the ISMRM‐NIST phantom. (A) Linear regressions. The black solid line is the identity line. (B) Bland Altman analyses showing the bias (thicker lines) and limits of agreement (LoA, thinner lines) for MOLLI (gray circles and continuous lines, 18.5 and −36.8 to 73.8 ms), 1FA (red squares and dotted lines, −39.6 and −187.3 to 108.2 ms) and 2FA (green triangles and dashed lines, 6.1 and −44.3 to 56.6 ms).

#### 3.1.2 Trajectory Optimization

When analyzing the mean PSF across contrast bins and orientations, the lowest SPR and S_SD_PR were obtained with the T2B trajectory variation (0.023 and 0.0042, respectively) closely followed by the T2B‐GS (0.024 and 0.0043, respectively, *p =* 0.2 and *p =* 0.5). When visually observing the PSF, larger artifacts could be observed when not applying the golden‐step shuffling (Figure 6A–D, arrows).

**FIGURE 6:**
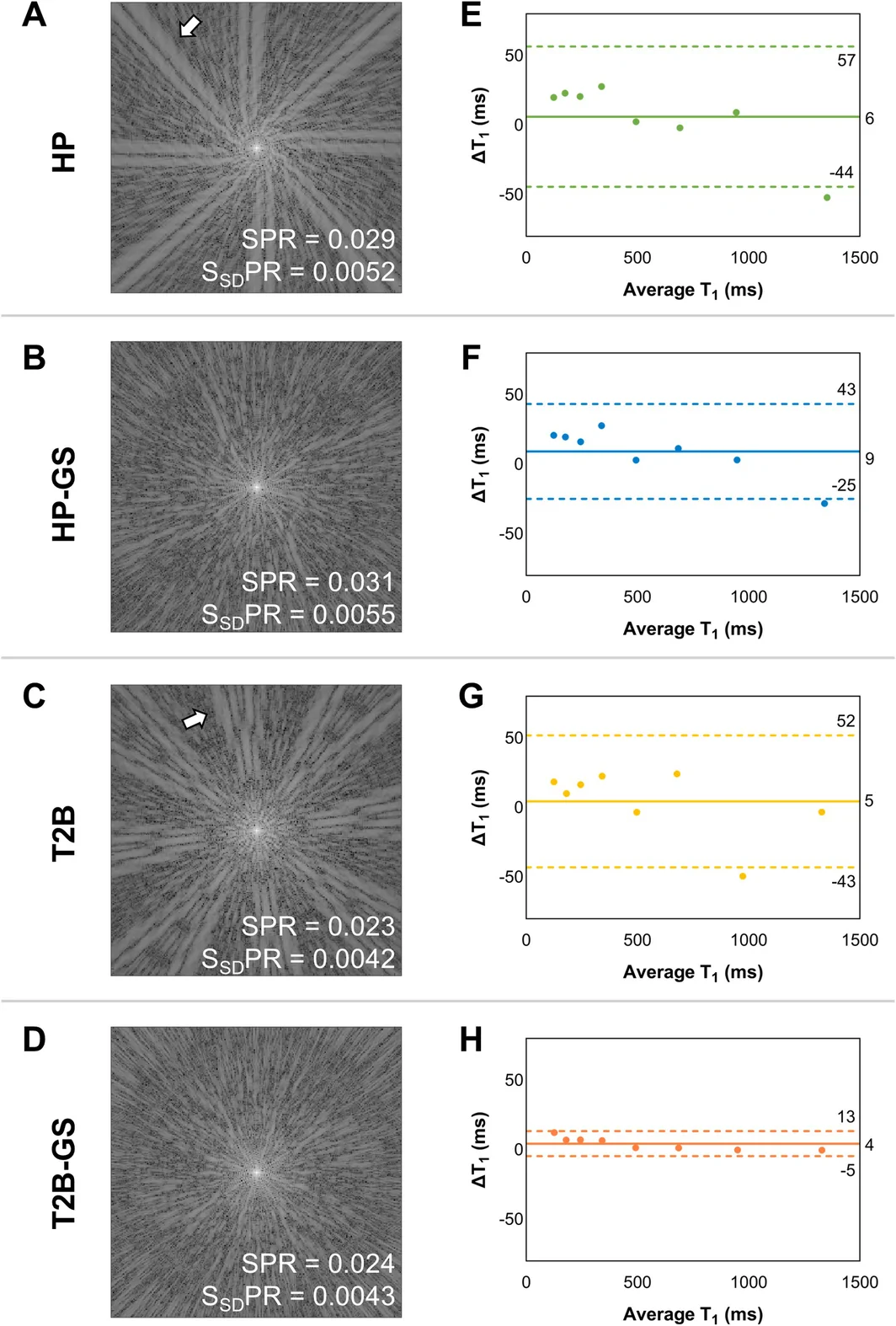
Comparison of the trajectory variations in the ISMRM‐NIST phantom. (A–D) PSF of the XY plane in the 15th contrast bin. Structured noise can be observed when no golden‐step shuffling (GS) is applied (arrows). The side‐lobe‐to‐peak ratio (SPR) and the ratio of the standard deviation of the side lobe magnitudes to the main peak magnitude (S_SD_PR) are indicated in the lower right corners. (E–H) Bland Altman plots of the estimated T_1_ values compared to a standard 2D IR‐SE sequence. Bias (continuous line) and confidence intervals (dashed line) values are indicated in the right part of the graphs.

The lowest RMSE was obtained with the T2B‐GS trajectory (RMSE = 4.8 ms). It also resulted in the narrowest confidence intervals (−5 to 13 ms, Figure 6H) compared to other trajectory variations (−44 to 57 ms for HP, −25 to 43 ms for HP‐GS and −43 to 52 ms for T2B, Figure 6E–G). This trajectory variation was therefore retained for the in vivo study.

### 3.2 In Vivo Imaging

The self‐gated respiratory signal was strongly correlated with the respiratory bellows signal (*r* = 0.82) and allowed for the estimation of T_1_ values in the different respiratory phases. The acquisition efficiency was 32% for CARISMAT1C. End‐expiratory (1185 ± 42 ms, Figure 7) and end‐inspiratory (1187 ± 43 ms) CCA T_1_ values were similar, and more precise compared to the second (1199 ± 63 ms) and third (1192 ± 44 ms) respiratory bins, and the non‐motion‐resolved T_1_ values (1206 ± 70 ms, *p >* 0.05). In addition, the scan‐rescan analysis revealed lower CoV for the end‐expiratory T_1_ values (1.6%) compared to those of the end‐inspiration (2.5%), and the second (4.9%) and third (4.6%) respiratory bins. As end‐expiration corresponds to the most quiescent respiratory phase [32], end‐expiratory T_1_ maps were retained for subsequent analysis.

**FIGURE 7:**
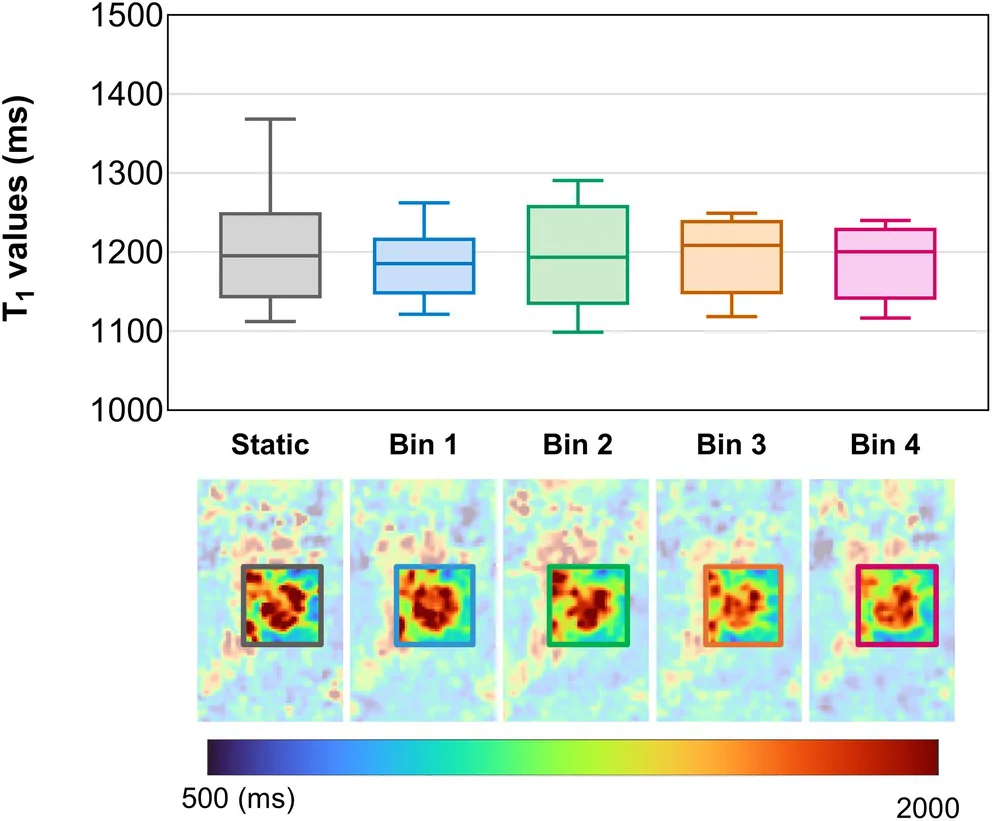
Motion‐resolved analysis of carotid vessel wall T_1_ maps. T_1_ values measured in the common carotid vessel wall from the static, non‐motion‐resolved reconstruction and from the respiratory‐resolved reconstruction. In the respiratory‐resolved approach, Bin 1 corresponds to end‐expiration and Bin 4 to end‐inspiration. Representative carotid T_1_ maps are shown below. End‐expiration images exhibit reduced geometric distortion due to improved alignment of the source images, resulting in better visualization of the carotid artery.

When using the 2D black‐blood image as a delineation template, end‐expiratory CCA vessel wall T_1_ values were slightly higher (1185 ± 42 ms, Figure 8A, Figure 9C–F) than MOLLI values (1162 ± 84 ms, *p =* 0.37), with the former showing less intersubject variability. In the sternocleidomastoid muscle, no difference was found between the 2FA T_1_ map (1051 ± 68 ms) and MOLLI (1064 ± 40 ms, *p =* 0.5). Co‐registered B_1_^+^ maps had lower values in the carotid blood pool (Figure 9A,B), which compared to the static tissues experiences fewer RF pulses as a consequence of inflow.

**FIGURE 8:**
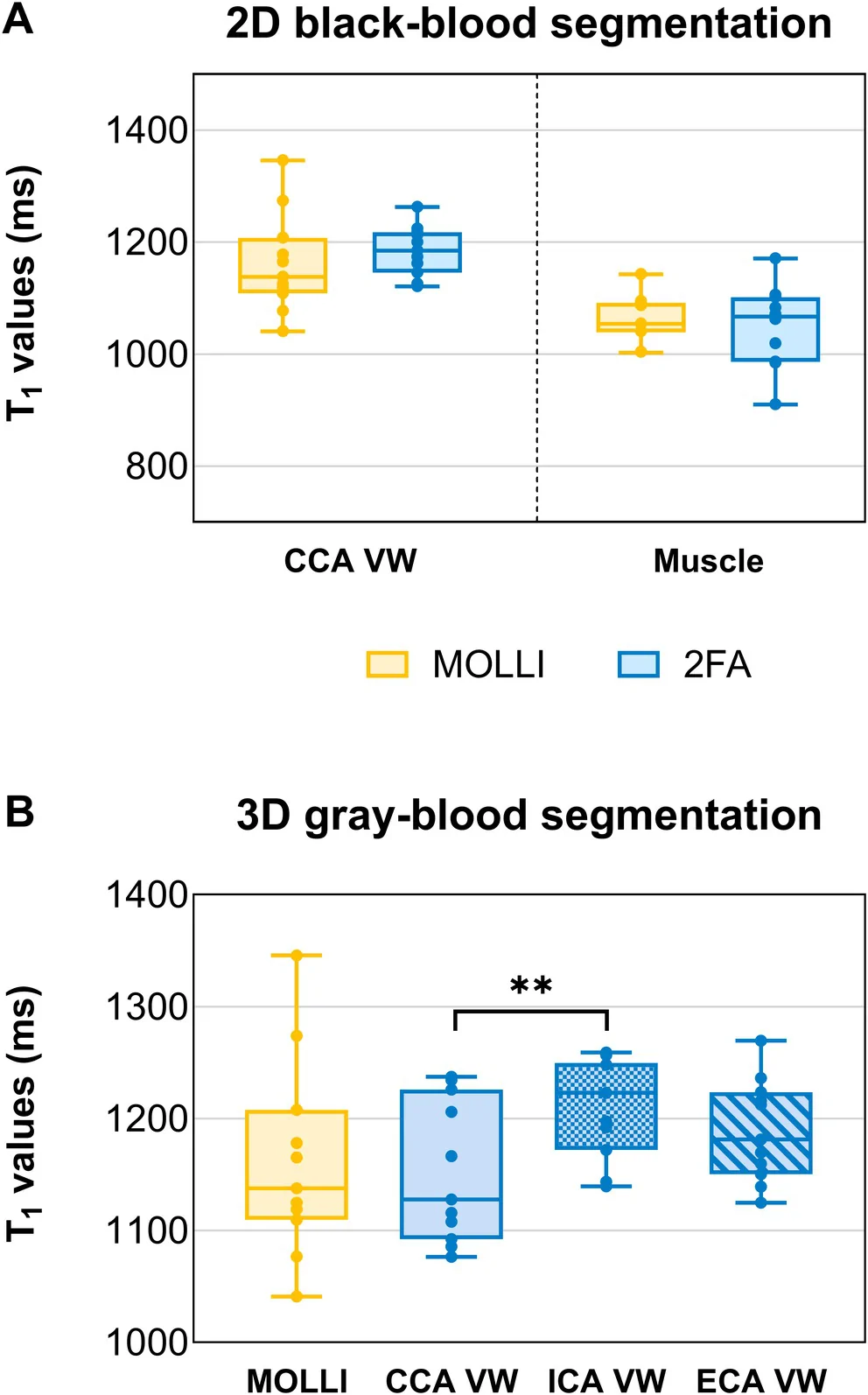
Regional in vivo T_1_ values. (A) T_1_ values in the common carotid artery vessel wall (CCA VW), muscle and blood when using the 2D black‐blood image as delineation template. There were no significant differences between MOLLI and the 2FA for the CCA VW and muscle (*p* = 0.37 and *p* = 0.5 respectively). (B) T_1_ values obtained when using a 3D gray‐blood image in the CCA, internal (ICA) and external (ECA) carotid arteries. All T_1_ values correlated well with MOLLI across all arterial sections and resulted in significant differences only for the CCA vs. ICA segment (*p* = 0.004).

**FIGURE 9:**
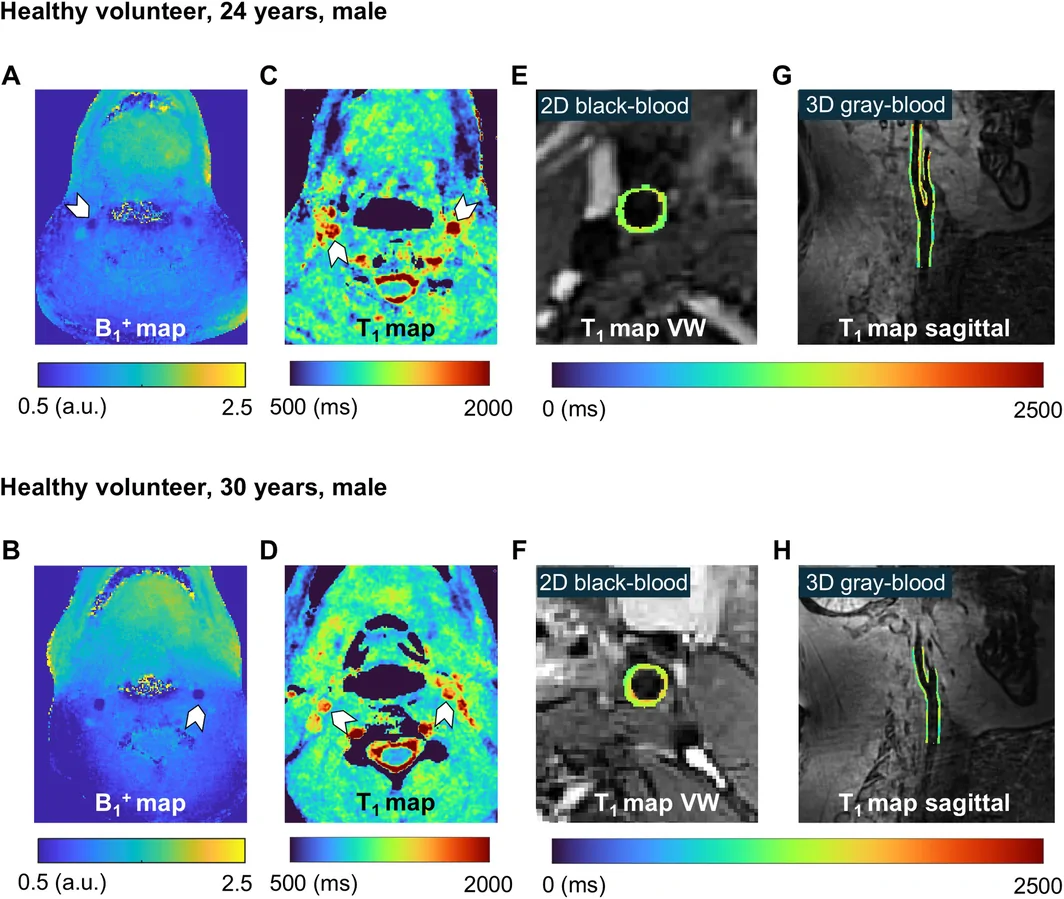
In vivo maps from two healthy volunteers. White arrows indicate carotid arteries. (A–D) B_1_^+^ and T_1_ maps of the neck. In (E,F) the T_1_ map from the vessel wall (VW) is overlaid on the 2D black‐blood image. In (G,H) the sagittal VW T_1_ map is overlaid on the synthetic 3D gray‐blood image.

For the repeatability assessment, the interscan vessel wall T_1_ CoV was lower for the 2FA technique (1.6%, *p =* 0.28) than for MOLLI (7.0%, *p =* 0.86). For the muscle, the T_1_ CoV was slightly higher with the 2FA technique (5.2%, *p =* 0.25) compared to MOLLI (4.5%, *p =* 0.92). Bland–Altman analyses revealed narrower LoA for both vessel wall (−53 to 95 ms) and muscle (−196 to 103 ms) when using 2FA compared to MOLLI (−304 to 279 ms and −170 to 284 ms, respectively).

The CCA, ICA, and ECA vessel walls could consistently be segmented using the 3D gray‐blood image as a guide (Figures 8B and 9G,H). No significant T_1_ differences were observed when comparing the MOLLI CCA T_1_ values (1162 ± 84 ms) to those of the proposed technique in the CCA (1152 ± 60 ms, *p =* 0.8), ICA (1206 ± 42 ms, *p =* 0.2), or ECA (1181 ± 37 ms, *p =* 0.4). Post hoc paired testing with correction for multiple comparisons resulted in significant differences only for the CCA vs. ICA segment (*p =* 0.004).

## 4 Discussion

We successfully developed and validated a new carotid T_1_ mapping technique. It was optimized through numerical simulations and phantom studies, and its precision and repeatability were demonstrated in vivo. 3D carotid artery vessel wall T_1_ maps using a 2FA technique were successfully obtained in all participants. T_1_ value overestimation was successfully avoided when including B_1_^+^ in the signal model, as shown by the high agreement of the 2FA with IR‐SE maps. Binning‐related artifacts, geometrical distortions, and structured noise in the images caused by coherent undersampling patterns were successfully reduced by using a top‐to‐bottom phyllotaxis trajectory and adding a golden‐step shuffle to the polar angles of the spiral interleaves.

To optimize and characterize the 2FA technique, phantom experiments were conducted. B_1_^+^ values within the spheres were lower than nominal, which led to the T_1_ overestimation when using the uncorrected 1FA technique (i.e., the lower apparent flip angle, the higher apparent T_1_). After applying a 2FA technique in a phantom, Serry et al. [6] reported a reduction of the bias (from −63 to −21 ms) along with narrower confidence intervals (from −120 to −6 ms, to −31 to −11 ms). In our 2FA phantom scans, the bias and confidence intervals were reduced even further after optimizing the trajectory to a top‐to‐bottom phyllotaxis with golden‐step shuffling of the shots, yielding a bias of 4 ms and confidence intervals of −5 to 13 ms. By simulating the complete magnetization evolution in the dictionary, the Look‐Locker effect was correctly modeled [33], and T_1_ underestimation was avoided. Phantom experiments with other T_1_ mapping techniques often showed larger discrepancies than our technique compared with 2D IR‐SE reference values in similar T_1_ ranges: the GOAL‐SNAP [5] study, for example, reported a bias of −29 ms with confidence intervals of −111 to 60 ms, while the SIMPLE technique [34] also resulted in phantom T_1_ confidence intervals that were wider than those obtained in this study. Similarly, when using a multi‐echo phase‐sensitive inversion‐recovery T_1_ mapping sequence [35], an average difference of 24 ms and limits of agreement ranging from −160 to 111 ms were shown, while approaches based on deep‐learning reconstruction and fitting [36] exhibited broader confidence intervals and higher bias than those reported in this study.

The use of quantitative imaging to characterize atherosclerotic plaque components in the carotid artery [37] goes beyond current clinical decision making, which relies largely on stenosis severity and largely ignores plaque composition despite its impact on plaque vulnerability [3]. There is currently no consensus on the “true” T_1_ value of the healthy carotid vessel wall, as evidenced by prior 3D T_1_ mapping studies [5, 34, 36, 38, 39, 40, 41], which reported a wide range of values, from 743 to 1212 ms at 3 T. Notably, the T_1_ values obtained in our study (1185 ± 42 ms) fall well within this reported range while demonstrating lower intersubject variability than most of the aforementioned studies (intersubject standard deviation ranging from 40 to 136 ms), highlighting the robustness and consistency of our approach. When analyzing T_1_ values of the sternocleidomastoid muscle, the spread was smaller than in the carotid vessel wall (from 1027 to 1191 ms) [5, 34, 35, 38, 39, 40, 41]. Our muscle values also fell within this range (1039 ± 60 ms). If any was applied, most of these studies relied on a separate B_1_^+^ mapping acquisition to correct for B_1_^+^ inhomogeneities. In this study, the B_1_^+^ correction was included in the fitting procedure thanks to the 2FA technique, where dictionary entries were already simulated for both flip angles using EPG, producing a B_1_^+^ map co‐registered to the T_1_ map.

The continuous free‐running data acquisition enabled the retrospective reconstruction of a synthetic 3D gray‐blood image, which in turn allowed for the segmentation and measurement of vessel wall T_1_ values across different regions of the carotid artery tree. The 2D black‐blood image was only used to measure the T_1_ and B_1_^+^ values of the ROIs once as an initial calibration. Although only one gray‐blood image was reconstructed to optimize the vessel wall depiction by selecting k‐space lines corresponding to the black nulling and maximum vessel‐to‐muscle contrasts, theoretically, any contrast of interest could be generated by selecting the k‐space lines corresponding to the TI of interest. In a follow‐up study, this could be compared to a clinical routine multi‐contrast 2D protocol, which is characterized by long scan times and misregistration of the images generated by the TOF, T_1_‐weighted, and T_2_‐weighted sequences [42, 43].

This study has several limitations. First, no patients with carotid atherosclerotic plaques were included, and future work will focus on the validation of the technique in patients. However, considering the phantom experiments and the good agreement between our 2FA technique and the IR‐SE sequence, we hypothesize that T_1_ values corresponding to intraplaque hemorrhage (200–650 ms) [5, 40, 41], lipid‐rich necrotic core (1181 ms) [41] and fibrous tissue (1316 ms) [41] should be differentiable using this method. Secondly, as a consequence of the respiratory‐resolved approach, three quarters of the already undersampled data were discarded after reconstruction by only preserving the end‐expiratory T_1_ map, which presented less intersubject variability, as previously reported [7]. A solution to this inconvenience could be to incorporate an adapted 3D non‐rigid intra‐bin motion correction approach already successfully applied in cardiac MRI [44], which does not discard any data, thus improving the quality of the source images and the final T_1_ maps, reducing the scan time. Although inflow effects were mitigated through the use of non‐selective inversion pulses and B_1_^+^ correction, they were not explicitly modeled in the EPG simulations. Consequently, blood T_1_ values may be underestimated. Future work should investigate the impact of inflow effects more comprehensively and assess their influence on gray‐blood image generation and contrast optimization. Lastly, T_2_ values could not be measured as no T_2_ preparation modules were included in the acquisition scheme. T_2_ mapping is particularly useful when assessing lipid‐rich necrotic core, whose T_1_ values are very similar to the ones of healthy carotid vessel wall [41, 45]. To implement this in the 2FA technique, sequence optimization should be performed to make a compromise between the amount of data acquired and a reasonable acquisition time.

## 5 Conclusion

After optimization in numerical simulations and a phantom, the 2FA free‐running technique CARISMAT1C was successfully used to simultaneously generate high‐resolution B_1_^+^ and T_1_ maps in the carotid vessel walls of all participants. CARISMAT1C significantly increased T_1_ accuracy in vitro, outperforming the 1FA technique and MOLLI. The 2FA sequence with an optimized top‐to‐bottom with golden‐step shuffling scheme further improved the agreement between the 2FA T_1_ values and the IR‐SE reference. In healthy volunteers, end‐expiratory T_1_ maps showed low intersubject variability, and the internal, external, and common carotid artery vessel wall T_1_ values could be measured thanks to the reconstruction of a 3D‐gray blood image from the raw k‐space data.

## Funding

This work was supported by Schweizerischer Nationalfonds zur Förderung der Wissenschaftlichen Forschung, 32003B_182615, CRSII5_202276, FNS_310030_215604, PZ00P3_202140 and Schweizerische Herzstiftung, FF23129.

## Conflicts of Interest

The authors declare no conflicts of interest.

## Data Availability

The data that support the findings of this study are available on request from the corresponding author. The data are not publicly available due to privacy or ethical restrictions.
